# Evaluation of an electron Monte Carlo dose calculation algorithm for electron beams

**DOI:** 10.1120/jacmp.v9i3.2720

**Published:** 2008-06-23

**Authors:** Ye Angela Hu, Haijun Song, Zhe Chen, Sumin Zhou, Fang‐Fang Yin

**Affiliations:** ^1^ Department of Radiation Oncology University of Colorado Health Science Center Aurora CO; ^2^ Department of Radiation Oncology Duke University Medical Center Durham NC; ^3^ Department of Therapeutic Radiology Yale University School of Medicine New Haven CT USA

**Keywords:** electron Monte Carlo, commissioning, verification, dosimetry, radiation

## Abstract

The electron Monte Carlo (eMC) dose calculation algorithm of the Eclipse treatment planning system is based heavily upon Monte Carlo simulation of the linac head and modeling of the linac beam characteristics with minimal measurement of beam data. Commissioning of the eMC algorithm on multiple identical linacs provided a unique opportunity to systematically evaluate the algorithm with actual measurements of clinically relevant beam and dose parameters. In this study, measured and eMC calculated dose distributions were compared both along and perpendicular to electron beam direction for electron energy/applicator/depth combination using measurement data from four Varian CLINAC 21EX linear accelerators (Varian Medical Systems, Palo Alto, CA). Cutout factors for sizes down to 3×3 cm were also compared. Comparisons between the measurement and the eMC calculated values show that the $R_{90}$, $R_{80}$, $R_{50}$, and $R_{10}$ values mostly agree within 3 mm. Measure and calculated bremsstrahlung dose Dx correlates well statistically although eMC calculated $D_{x}$ values are consistently smaller than the measured, with maximum discrepancy of 1% for the 20 MeV electron beams. Surface dose agrees mostly within 2%. Field width and penumbra agree mostly within 3 mm. Calculation grid size is found to have a significant effect on the dose calculation. A grid size of 5 mm can produce erroneous dose distributions. Using a grid size of 2.5 mm and a 3% accuracy specified for the eMC to stop calculation iteration, the absolute output agrees with measurements within 3% for field sizes of 5×5 cm or larger. For cutout of 3×3 cm, however, the output disagreement can reach 8%. Our results indicate that the eMC algorithm in Eclipse provides acceptable agreement with measurement data for most clinical situations. Calculation grid size of 2.5 mm or smaller is recommended.

## I. INTRODUCTION

The electron Monte Carlo (eMC) algorithm of the Eclipse treatment planning system (Varian Medical Systems, Palo Alto, CA) uses electron energy dependent dose kernel libraries, of macroscopic spheres of various radii and materials, that are pre‐calculated with the EGS4 Monte Carlo code.^(1)^ The advantage of this macro‐Monte Carlo approach is short treatment planning time with dose calculation accuracy similar to that of bona fide Monte Carlo program. Explicit knowledge of beam forming parts inside a Varian CLINAC 21EX linear accelerator is used in pre‐calculating these dose kernels. Therefore, minimal amount of measured beam data is required for the commissioning of the eMC treatment planning system. The required input beam data include the profiles in air at 95 cm for each energy, the relative depth‐dose curve in water and absolute dose at a specified point for each energy/applicator combination. Profiles at different depths in water for each electron energy/ applicator combination are not used as input. The algorithm uses initial phase space model^(2)^ to derive the initial beam phase space based on the PDDs, assuming a linac head design of a standard Varian Clinac 21EX linear accelerator.

Agreement between calculation and measurement can be affected by many factors. Previously published studies provided substantial information on factors related to eMC calculation settings, such as accuracy, calculation grids and smoothing methods using measurement data from one or two machines.^(3)^ However, because of the stochastic nature of Monte Carlo calculation and inevitable variations in measurements, comparisons based upon data from a group of machines provide more reliable results. Further more, systematic information on how other clinically relevant factors (such as electron energies, cone sizes, and depth) affect the agreement, is lacking. We have recently commissioned the eMC algorithm for four Varian Clinac 21EX linear accelerators at a single clinic location, which provide a unique opportunity to systematically compare eMC calculation and actual measurements. In addition to profiles at various depths, electron cutout factors for cutouts down to the size of 3×3 cm were measured. The output verification is especially important due to the stochastic nature of the Monte Carlo method which gives the output with a specified statistical uncertainty.

We present a systemic comparison between eMC calculations and extensive measurements on four Varian Clinac 21EX accelerators. Recommendations on how to use the treatment planning systems are given.

## II. MATERIALS AND METHODS

The four commissioned linear accelerators share the same Varian Clinac 21EX model number. Their photon beams are matched in PDD and beam profiles. The electron beams are not matched.

Required beam data for the eMC algorithm were collected for each of the machines. Profiles in air were obtained for each electron energy at 95 cm. A large water tank, 48×48×41 cm, with a 3D scanning mechanism (Blue Phantom: Scanditronix‐Wellhofer, Bartlett, TN) was used to acquire relative depth‐dose curve in water at SSD=100 cm and absolute dose depth of 100% dose. Both the reference and field detectors were diodes. The distance of 0.05 cm from the active layer to the casing surface of the scanning diode was taken into account. From PDDs, the following parameters were derived: $R_{100}$ (depth of 100% dose), $R_{90}$ (depth at which dose is 90% of the maximum dose), $R_{50}$, $D_{x}$ (bremsstrahlung tail) and surface doses. Profiles were scanned at the depths of $R_{100}$, $R_{90}$, and $R_{50}$ in water for each beam energy and applicator combination. The scanning system was set to acquire data points every 0.5 mm. OmniPro Accept (Version 6.1, Scanditronix‐Wellhofer, Bartlett, TN) was used to acquire and export the data. Square Cerrobend electron cutouts were made for each applicator down to the size of 3×3 cm. For consistency, all cutout factors were measured with the same mini ionization chamber CC01 (cavity volume 0.01 cm^3^, radius 1.0 mm, Wellhofer‐Scanditronix, Bartlett, TN) placed at $R_{100}$ of each energy/cone. Data were collected by different groups of physicists using the same measurement devices.

Measured beam data acquired from phantom scan were exported from OmniPro in ASCII format. Each measurement setup was then calculated in Eclipse eMC and the PDD and profiles were exported from Eclipse in the DICOM format and converted to ASCII format for comparison. Linear interpolation of the eMC dose distribution was used when necessary to match the measurement locations.

Dose distributions for each energy/cone combination were calculated with algorithms commissioned for each machine. The accuracy was set at 2%. The software allows the planner to define either statistical accuracy or numbers of particles to transport. According to the manufacturer, the accuracy refers to the mean statistical error in dose for all voxels receiving more than 50% of the maximum dose value located within the region of interest.^(7)^ When the stated mean statistical accuracy is achieved, the algorithm will stop simulation. Medium level 3‐D Gaussian method was used for smoothing after calculation. Calculation grid size of 2.5 mm was used throughout except for a field of 3×3 cm, where 1 mm was used and will be discussed in the grid size investigation. Selection of these parameters was based upon computational time and published studies.^3^, ^5^ Each electron cutout configuration was calculated in Eclipse and the cutout factors were derived from the calculations. A total dose of 10,000 cGy was prescribed and normalized to $R_{100}$. Since different conventions are being used for some of the beam parameters, the definitions, as used in this study, are listed in Appendix A for clarity.

The combination of energy/cone size/depth/cutout size in measurement and eMC calculation generated a substantial amount of data. In order to analyze the data systematically and consistently, a statistical software package SAS (SAS, Cary, NC) was utilized. Analysis of variance (ANOVA) and linear regression were used to study the influence of factors (e.g., machines, energies, cone sizes) on the discrepancies between measurements and eMC calculation results. Significant level was set at 0.05.

## III. RESULTS AND DISCUSSION

### A. Central‐axis depth dose curve

#### 1. Depth in water

**Figure 1:**
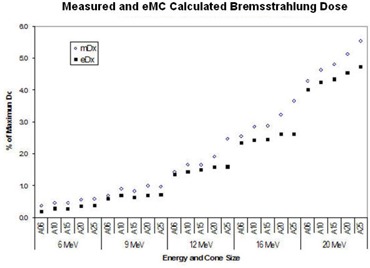
Comparison of measured and eMC‐calculated depths in water. (a) Discrepancy of measured depth, (b) Discrepancy of eMC calculated depth, (c) Difference between eMC and measured depth

Table 1 compares the depth $R_{90}$, $R_{80}$, $R_{50}$, and $R_{10}$ in water of measured and eMC calculated PDD at 90%, 80%, 50% and 10% of maximum dose, averaged over four tested machines. $R_{100}$ was not compared because the PDD curve around $R_{100}$ is fairly flat therefore a small amount of noise in PDD measurement can cause a big error in identifying the exact location of the PDD maximum. Small differences in each machine's energy, scatter foils or scanning system setup can affect these parameters.^(6)^ Nonetheless, ANOVA analysis indicated that machine was not a statistically significant factor (p< 0.05) affecting the difference between the measured and eMC calculated results. Further analyses shown in Figs. 1a – 1c revealed a few interesting findings. Fig. 1a illustrates the range of disagreement in measured depths among the machines. For cones smaller than 20×20 cm, the difference was mostly smaller than 2 mm. These differences were comparable with those in the literature. ^8^, ^9^ A maximum of 4 mm difference was observed for the 25×25 cm cone at 10% dose for 16 MeV and 20 MeV. Disagreement among eMC calculated depths is shown in Fig. 1b. Variations of depth in water at 10% dose among machines were rarely reported in literature because this range is contaminated with bremsstrahlung photons and beyond the range of clinical interest. Nevertheless we report our result here for interested readers. Our result indicates that even though $R_{50}$, $R_{80}$ and $R_{90}$ agreed reasonably well among machines, larger variations at $R_{10}$ are possible, likely due to slight variations in energy spectrum. Contrary to measurement, the largest differences in eMC calculated depths occurred at smallest cone (6×6 cm) with a magnitude of 5–7 mm at lower energy of 6 MeV and 9 MeV (Fig. 1b). Because our machines were individually measured and commissioned using each machine's own measurement data, the differences we observed could be a combination of variations in inherent beam spectrum, measurement uncertainty and statistical uncertainty of the algorithm.

**Table 1:** Measured and eMC calculated depths (mm) averaged over four Varian Clinac 21EX

		*6 MeV*		*9 MeV*		*12 MeV*		*16 MeV*		*20 MeV*	
*Cone*	*Depth*	*Measured*	*eMC*	*Measured*	*eMC*	*Measured*	*eMC*	*Measured*	*eMC*	*Measured*	*eMC*
A06	R10	29.3	26.2	43.7	43.3	60.5	60.8	80.4	80.3	102.5	101.5
A06	R50	24.0	21.6	36.3	35.5	50.5	50.5	66.1	66.1	81.2	81.1
A06	R80	20.2	19.6	31.0	30.7	43.2	43.2	55.1	55.4	64.1	64.0
A06	R90	18.5	18.2	28.5	28.1	39.5	39.7	49.2	49.7	54.5	54.4
A10	R10	29.3	31.4	43.7	45.2	60.6	61.0	80.6	80.5	103.5	101.5
A10	R50	24.0	24.8	36.3	36.6	50.5	50.4	66.7	66.4	83.7	82.7
A10	R80	20.3	20.6	31.0	31.0	43.3	42.9	56.6	56.0	68.5	67.6
A10	R90	18.4	18.6	28.4	28.4	39.7	39.5	51.2	50.2	59.0	58.3
A15	R10	29.3	31.4	43.7	45.0	60.6	60.9	80.7	80.6	104.0	101.8
A15	R50	24.0	24.8	36.3	36.7	50.6	50.3	66.8	66.3	84.1	82.8
A15	R80	20.2	20.6	31.0	31.1	43.4	42.7	56.8	56.2	69.3	67.7
A15	R90	18.4	18.7	28.5	28.6	39.7	39.1	51.5	50.5	60.1	58.4
A20	R10	29.4	31.4	43.9	45.0	60.9	61.4	81.0	80.7	104.5	102.3
A20	R50	24.1	24.8	36.5	36.8	50.7	50.9	67.0	66.6	84.3	83.4
A20	R80	20.3	20.6	31.1	31.3	43.5	43.3	56.9	56.7	69.4	68.6
A20	R90	18.5	18.7	28.5	28.6	39.9	39.6	51.5	50.9	60.4	59.4
A25	R10	29.5	31.5	44.0	45.4	61.3	61.6	81.5	80.8	105.3	102.5
A25	R50	24.2	24.8	36.6	36.9	50.9	50.9	67.3	66.8	84.7	83.9
A25	R80	20.4	20.7	31.3	31.4	43.7	43.3	57.2	56.7	69.9	69.2
A25	R90	18.6	18.7	28.7	28.8	40.0	39.5	51.7	50.9	61.0	61.0

Differences between measured and eMC calculated $R_{90}$, $R_{80}$, $R_{50}$, and $R_{10}$, averaged over machines, were calculated and shown in Fig. 1c. Larger discrepancies between the measurement and eMC calculated result occurred at $R_{10}$. Overall, the discrepancies were mostly within 3 mm. Electron beam energy appeared to affect the discrepancies, too. At lower energy levels (6 and 9 MeV), the eMC calculated $R_{90}$, $R_{80}$, $R_{50}$, and $R_{10}$ were slightly larger than measurement, indicating that eMC calculated PDDs shifted to the right of the measured PDD. Measurement and eMC calculation agreed reasonably well at 12 MeV and 16 MeV. As beam energy went up to 20 MeV, eMC calculation became slightly smaller than measurement indicating the eMC calculated PDDs shifted to the left of the measured PDD. As the end user of the software, it is difficult to know the reason for the above observation without knowing the details of parameters and procedures of the algorithm. Nonetheless we thought the observation was interesting enough to be reported here.

#### 2. Dose due to Bremsstrahlung

**Figure 2:**
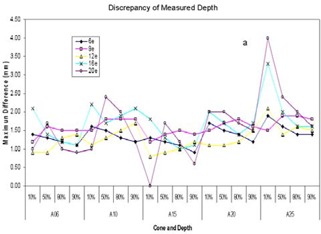
Measured and eMC‐calculated Bremsstrahlung tails.

Linear regression analysis indicated that eMC calculated $D_{x}s$ correlated well with measured $D_{x}s$ (R‐square=0.95, p<0.01). However, compared to measurement, eMC calculated $D_{x}s$ were consistently smaller than measured $D_{x}s$ except for a few outliers. Fig. 2 summarizes the measured and eMC calculated $D_{x}$ averaged over four machines. In both measurement and calculation, $D_{x}$ increased with energy and cone size, reaching 5–6% at 20 MeV. The discrepancy between measurement and calculation became more apparent for larger cone sizes at higher energies, reaching about 1%.

#### 3. Surface dose

**Figure 3:**
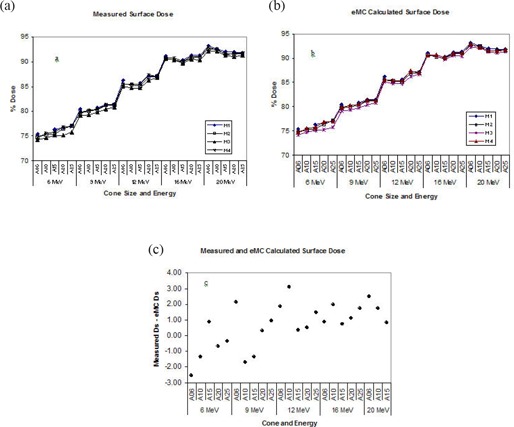
Measured and eMC‐calculated surface doses for four Varian 21EX linear accelerators. (a) Measured surface doses, (b) eMC‐calculated surface doses, (c) Differences between measured and eMC‐calculated surface doses

Surface dose for both measurement and eMC calculation was determined at 0.5 mm depth on central‐axis PDD curves. As shown in Fig. 3a and Fig. 3b, surface dose increases with electron beam energy, averaging around 75% at 6 MeV and around 90% at 20 MeV. Variations among the machines were small for both measurements (Fig. 3a) and eMC calculation (Fig. 3b). Discrepancies between measurement and eMC were mostly within 2% with slightly larger value (yet still within 3%) for 6×6 cm cone (Fig. 3c).

### B. Dose distributions

#### 1. Field width

**Figure 4:**
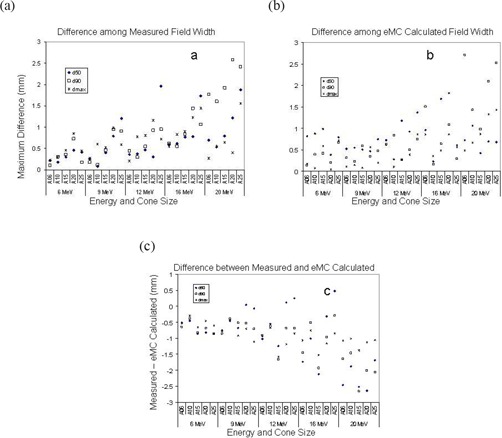
Comparison of measured and eMC‐calculated field widths. (a) Differences among measured field widths, (b) Differences among eMC‐calculated field widths, (c) Differences between measured and eMC‐calculated field widths.

Table 2 lists measured and eMC calculated field width of crossplane profiles acquired at $R_{100}$, $R_{90}$, and $R_{50}$, averaged over the machines. Similar results were obtained between crossplane and inplane from eMC calculation, we therefore only present results from crossplane for presentation clarity. Because difference between measured and eMC calculated field width can be affected by measurement differences or statistical variations of eMC calculation, we further analyzed these factors, as shown in Figs. 4a – 4c. Fig. 4a shows the measured differences among the four machines. When energy increased, the disagreement range (maximum of measurement – minimum of measurement) among the machines increased as well, reaching a maximum of 2.6 mm at 20 MeV. Similar pattern was seen for eMC calculated field width, with a comparable maximum at 20 MeV (Fig. 4b). However, when comparing each machine's measured field width with its corresponding eMC calculated field width, one can observe that the measured field width was systematically smaller than eMC calculated field width in most cases. Higher energies (16 MeV and 20 MeV) appeared to have larger discrepancies than lower energies (6 MeV, 9 MeV and 12 MeV) as shown in Fig. 4c. Overall, however, the discrepancy between eMC calculated and measured field width were all within 3 mm. For lower and medium energies (6–16 MeV) and small cone sizes (<20×20 cm) which are most frequently encountered in clinic, the agreements were mostly within 1 mm.

**Table 2:** Measured and eMC calculated field width (mm) of crossplane profiles acquired at $d_{\text{max}}$, $R_{90}$ and $R_{50}$, averaged over four Varian Clinac 21EX

		*6 MeV*		*9 MeV*		*12 MeV*		*16 MeV*		*20 MeV*	
*Cone*	*Depth*	*Measured*	*eMC*	*Measured*	*eMC*	*Measured*	*eMC*	*Measured*	*eMC*	*Measured*	*eMC*
6.0	R50	60.6	61.2	61.5	62.3	62.6	63.6	64.4	66.2	67.1	69.6
10.0	R50	101.7	102.2	103.4	103.8	104.7	105.2	106.7	107.7	108.7	110.5
15.0	R50	152.2	153.1	154.7	155.3	156.3	157.5	159.0	161.1	161.4	163.9
20.0	R50	203.5	204.3	206.5	206.4	209.6	209.5	213.3	213.6	215.9	218.5
25.0	R50	254.4	255.3	257.7	257.8	261.6	261.4	266.4	265.9	269.8	271.5
6.0	R90	60.8	61.5	61.7	62.5	62.6	63.5	64.2	65.6	65.4	67.0
10.0	R90	102.1	102.5	103.7	104.1	105.1	105.7	106.9	107.4	108.0	109.4
15.0	R90	152.6	153.5	155.0	155.5	156.5	158.1	159.0	160.9	160.6	163.3
20.0	R90	204.1	204.8	206.8	207.3	209.8	210.5	213.0	214.0	214.8	216.8
25.0	R90	255.2	256.1	258.2	258.9	261.7	262.3	266.0	266.3	268.3	270.3
6.0	dmax	60.9	61.4	61.6	62.3	62.2	63.1	62.2	63.2	61.5	62.6
10.0	dmax	102.1	102.4	103.2	103.7	104.1	104.7	104.2	105.0	102.9	103.9
15.0	dmax	152.8	153.5	154.5	155.4	155.4	157.0	155.7	157.3	154.0	155.4
20.0	dmax	204.1	204.6	206.2	206.9	208.0	209.2	208.2	209.4	205.6	206.7
25.0	dmax	255.2	255.8	257.3	258.4	259.7	260.6	260.1	260.9	256.9	258.0

#### 2. Penumbra

**Figure 5:**
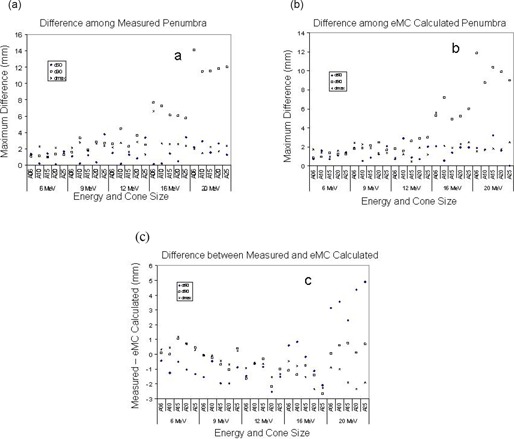
Comparison of measured and eMC‐calculated penumbras. (a) Differences among measured penumbras, (b) Differences among eMC‐calculated penumbras, (c) Differences between measured and eMC‐calculated penumbras.

Table 3 lists measured and eMC calculated penumbra of crossplane profiles acquired at $R_{100}$, $R_{90}$, and $R_{50}$, averaged over the machines. Compared to field width, the uncertainties in both measurement and eMC calculation were much larger, as shown in Fig. 5a and Fig. 5b. Here the machines demonstrated their “individuality”, with penumbra from one machine consistently smaller than the others. Nonetheless, the difference between measured and its corresponding eMC calculated penumbra was much smaller (Fig. 5c), a result of individual commissioning of each machine's algorithm using its own electron beam data. At clinically more relevant depth $\left(R_{90}\;\text{and}\;R_{100}\right)$, the difference between measured and eMC calculated penumbra was within 3 mm.

**Table 3:** Measured and eMC calculated penumbra (mm) of crossplane profiles acquired at $d_{\text{max}}$, $R_{90}$ and $R_{50}$, averaged over four Varian Clinac 21EX

		*6 MeV*		*9 MeV*		*12 MeV*		*16 MeV*		*20 MeV*	
*Cone*	*Depth*	*Measured*	*eMC*	*Measured*	*eMC*	*Measured*	*eMC*	*Measured*	*eMC*	*Measured*	*eMC*
6.0	R50	25.6	26.0	31.9	33.4	39.6	41.1	47.9	47.3	54.6	51.5
10.0	R50	25.9	27.1	32.3	32.7	41.0	41.6	50.8	49.9	60.6	57.1
15.0	R50	26.2	26.7	31.6	33.5	40.7	41.6	50.4	50.6	61.4	59.1
20.0	R50	26.3	27.3	32.2	34.1	40.0	42.5	49.7	50.8	61.7	57.3
25.0	R50	26.5	27.8	33.7	34.6	41.3	42.8	50.3	52.4	62.2	57.3
6.0	R90	25.4	25.3	30.6	30.7	36.7	38.3	41.3	42.4	42.0	41.9
10.0	R90	25.9	25.8	30.7	31.0	37.9	38.5	44.0	45.3	46.6	45.9
15.0	R90	26.4	25.3	30.6	31.3	38.1	38.4	44.1	44.9	46.9	46.1
20.0	R90	26.1	25.4	30.8	31.9	37.0	39.1	43.3	44.7	46.8	46.7
25.0	R90	26.1	25.7	32.2	31.8	38.0	39.0	43.3	46.0	47.1	46.3
6.0	dmax	21.7	21.4	23.7	23.8	24.8	25.7	18.7	19.1	12.0	12.9
10.0	dmax	22.1	21.6	24.0	24.0	25.7	26.3	20.6	21.3	12.3	13.3
15.0	dmax	22.5	21.3	23.8	24.2	26.0	26.6	20.5	22.1	12.1	14.0
20.0	dmax	22.2	21.6	24.0	24.6	25.1	26.6	20.0	22.3	11.9	14.3
25.0	dmax	22.0	21.7	24.5	24.3	25.7	27.1	20.1	22.3	11.8	13.7

#### 3. Agreement of relative dose

**Figure 6:**
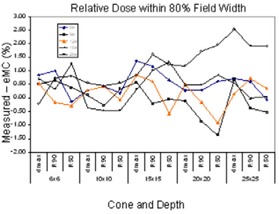
Relative doses within 80% field width.

We compared agreement of relative dose within 80% of field width. Differences between measured and eMC calculated relative dose in crossplane were used. Both measured and calculated dose are relative dose normalized to maximum dose at central axis. Measured profiles were smoothed, centralized, and made symmetric before exporting from OmniPro Accept, therefore the mean difference estimates the separation between the measured and calculated profiles. As shown in the Fig. 6, the differences were mostly smaller than 2% at 6, 9, 12 and 16 MeV. A maximum of 3% occurred at 20 MeV at $R_{100}$. These agreements demonstrate that the eMC calculated profiles follow the measured profiles very well given the dose accuracy of 2% set for the eMC calculations.

#### 4. Symmetry

**Figure 7:**
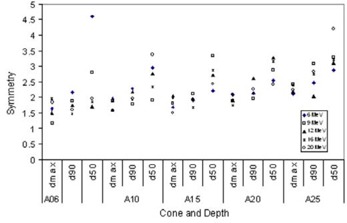
Symmetry of eMC‐calculated profiles

Symmetry for eMC calculated profiles at different depths for different energy/cone combinations is plotted in Fig. 7. Larger values were found for profiles at $R_{50}$. Symmetry for profiles at $R_{90}$ and $R_{100}$ were within 2.5% in general. The range of disagreement we observed here is reasonable, because the accuracy defined by the manufacturer refers to the mean statistical error in dose for all voxels receiving more than 50% of the maximum dose value located within the region of interest. With preset accuracy of 2%, outliers greater than 2% are expected, because the symmetry here is defined as the maximum difference in relative dose between points on equal distance from the central axis within the central 80% of the field width.

### C. Output factors (OF)

**Figure 8:**
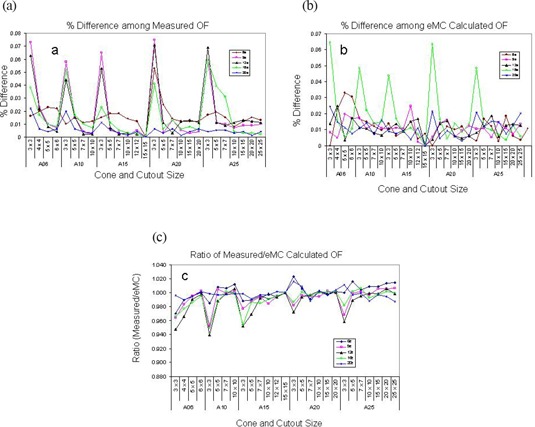
Measured and eMC‐calculated output factors (a) % differences among measured output factors, (b) % differences among eMC‐calculated output factors, (c) Ratio of measured to eMC‐calculated output factors.

Table 4 lists the measured and eMC calculated OFs averaged over four machines. Measured OFs and eMC calculated OFs agreed well for cutouts greater than 3×3 cm (Fig. 8c). The agreement is within 3% for cutouts greater than 5×5 cm, 5% for cutouts smaller than 5×5 cm but equal or larger than 4×4 cm. For cutout of 3×3 cm, however, the agreement was significantly poorer, reaching as much as 8%. We performed ANOVA analysis, which indicated that there were no statistically significant differences among the four machines (P<0.05). Energy level and cone size did not affect agreement either. Fig. 8a plots measured OFs among different machines. Up to 7.5% difference among machines was observed for 3×3 cm cutouts. Similar variations were observed for eMC calculated OFs among the machines (Fig. 8b). This indicates that large disagreement between OFs for 3×3 cm cutout can be contributed from both measurement and eMC uncertainties.

**Table 4:** Measured and eMC calculated cutout factors, averaged over four Varian Clinac 21EX

		*6MeV*				*9MeV*				*12MeV*				*16MeV*				*20MeV*			
*Cone*	*Cutout*	*Measured SD*	*eMC*	*SD*	*Measured*	*SD*	*eMC*	*SD*	*Measured*	*SD*	*eMC*	*SD*	*Measured*	*SD*	*eMC*	*SD*	*Measured*	*SD*	*eMC*	*SD*
A06	3×3	0.929	0.007	0.897	0.010	0.914	0.031	0.876	0.004	0.906	0.031	0.856	0.007	0.963	0.021	0.933	0.036	1.015	0.010	1.012	0.012
	4×4	0.971	0.009	0.958	0.014	0.972	0.010	0.954	0.003	0.965	0.012	0.933	0.011	0.994	0.010	0.973	0.008	1.029	0.003	1.020	0.007
	5×5	0.973	0.010	0.966	0.014	0.987	0.005	0.979	0.009	0.986	0.005	0.977	0.002	1.005	0.006	0.991	0.003	1.030	0.002	1.025	0.005
	6×6	0.970	0.010	0.968	0.008	0.987	0.003	0.988	0.008	0.989	0.002	0.986	0.008	1.000	0.009	0.999	0.005	1.026	0.003	1.029	0.003
A10	3×3	0.942	0.005	0.929	0.007	0.921	0.031	0.887	0.008	0.905	0.030	0.856	0.005	0.973	0.036	0.930	0.026	1.002	0.019	1.011	0.005
	5×5	1.003	0.008	1.009	0.005	0.994	0.008	0.999	0.005	0.992	0.010	0.982	0.005	1.007	0.010	0.996	0.012	1.035	0.003	1.033	0.006
	7×7	1.005	0.007	1.009	0.004	1.006	0.006	1.007	0.007	1.013	0.003	1.010	0.004	1.015	0.004	1.016	0.008	1.029	0.002	1.027	0.003
	10×10	0.997	0.007	1.008	0.006	1.005	0.003	1.006	0.005	1.010	0.004	1.013	0.004	1.017	0.005	1.013	0.007	1.026	0.003	1.023	0.008
A15	3×3	0.942	0.007	0.929	0.003	0.899	0.046	0.888	0.005	0.900	0.034	0.863	0.004	0.956	0.028	0.921	0.024	0.994	0.005	0.994	0.005
	5×5	1.011	0.009	1.000	0.004	1.010	0.006	0.994	0.005	0.996	0.007	0.967	0.007	1.005	0.005	0.990	0.008	1.017	0.002	1.008	0.003
	7×7	1.010	0.009	1.004	0.008	1.013	0.002	1.006	0.005	1.010	0.002	1.001	0.004	1.011	0.004	0.996	0.004	1.017	0.001	1.014	0.004
	10×10	1.004	0.007	0.994	0.002	1.008	0.001	0.992	0.011	1.009	0.001	1.007	0.007	1.010	0.001	1.000	0.005	1.011	0.001	1.008	0.002
	12×12	1.002	0.006	0.996	0.003	1.006	0.002	1.000	0.001	1.006	0.001	1.000	0.007	1.007	0.001	1.002	0.003	1.007	0.002	1.008	0.005
	15×15	1.000	0.000	1.000	0.000	1.000	0.000	1.000	0.000	1.000	0.000	1.000	0.000	1.000	0.000	1.000	0.000	1.000	0.000	1.000	0.000
A20	3×3	0.937	0.023	0.947	0.004	0.891	0.036	0.874	0.002	0.874	0.035	0.848	0.002	0.925	0.023	0.916	0.034	0.977	0.003	0.992	0.009
	5×5	1.015	0.011	1.022	0.006	0.990	0.005	0.984	0.007	0.975	0.005	0.966	0.006	0.982	0.003	0.981	0.009	0.992	0.002	1.000	0.002
	7×7	1.019	0.007	1.011	0.004	1.000	0.006	0.994	0.007	0.989	0.006	0.989	0.008	0.995	0.005	0.992	0.002	0.999	0.003	0.988	0.004
	10×10	1.016	0.005	1.017	0.005	0.998	0.003	0.992	0.003	0.994	0.005	0.995	0.003	0.997	0.003	0.997	0.007	0.997	0.001	0.998	0.003
	15×15	1.011	0.006	1.007	0.002	0.991	0.001	0.993	0.004	0.988	0.005	0.985	0.002	0.988	0.004	0.988	0.004	0.985	0.002	0.982	0.002
	20×20	1.008	0.006	1.007	0.001	0.988	0.002	0.986	0.005	0.981	0.005	0.981	0.003	0.982	0.002	0.983	0.007	0.976	0.003	0.978	0.003
A25	3×3	0.950	0.007	0.947	0.007	0.895	0.028	0.865	0.004	0.872	0.033	0.832	0.004	0.909	0.025	0.889	0.027	0.954	0.002	0.964	0.009
	5×5	1.008	0.009	1.024	0.006	0.966	0.009	0.968	0.005	0.949	0.009	0.941	0.005	0.947	0.018	0.953	0.005	0.968	0.003	0.964	0.005
	7×7	1.016	0.007	1.019	0.001	0.980	0.004	0.981	0.003	0.968	0.004	0.963	0.008	0.960	0.015	0.966	0.005	0.967	0.003	0.966	0.007
	10×10	1.011	0.004	1.018	0.008	0.977	0.004	0.973	0.007	0.969	0.005	0.967	0.002	0.970	0.002	0.964	0.003	0.968	0.002	0.956	0.006
	15×15	1.006	0.007	1.011	0.003	0.971	0.004	0.974	0.005	0.961	0.006	0.959	0.005	0.962	0.002	0.960	0.002	0.956	0.002	0.953	0.003
	20×20	1.001	0.008	1.012	0.002	0.966	0.004	0.970	0.003	0.955	0.005	0.959	0.006	0.953	0.002	0.955	0.006	0.947	0.001	0.941	0.006
	25×25	1.000	0.007	1.011	0.005	0.963	0.004	0.967	0.006	0.953	0.005	0.951	0.006	0.949	0.001	0.948	0.003	0.941	0.002	0.929	0.009

Reference cone size=15×15 cm

### D. Grid size

**Figure 9:**
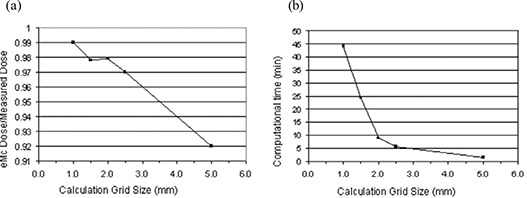
Calculation grid size, computational time and agreement, using data from 15×15 cm cone, 12 MeV. (a) Grid size vs. eMC/measurement agreement, (b) Grid size vs. calculation time

Investigation was conducted to study the impact of eMC calculation grid size on dose distribution calculation for a 15×15 cm cone at 12 MeV (Fig. 9a). Four dose calculation grid sizes, 1.0 mm, 1.5 mm, 2.0 mm. 2.5 mm, and 5.0 mm, were employed. Grid size of 5 mm gave a dose about 10% lower than actual measurement. Grid size of 2 mm provided significant improvement, with an eMC result being 3% lower than actual measurement. Changing grid size from 2 mm to 1.5 mm did not provide significant gain for this parameter until the grid size of 1 mm, yet it substantially increased computational time (Fig. 9b) on a typical computer of 2.66 GHz CPU (Xeon) and 3.3 GB RAM. Computational time was also energy dependent, for 15×15 cm cone with 2% accuracy and grid size 2.5 mm, 6 MeV required 2 minutes whereas 16 MeV required 8 minutes.

**Figure 10:**
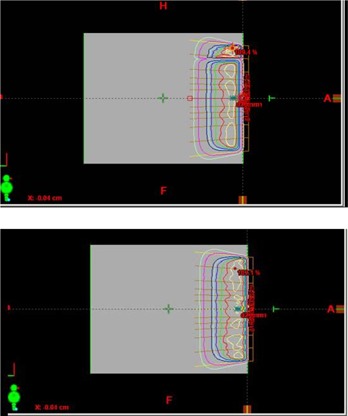
An example of hot spots and slits observed at calculation grid size of 5 mm for cone size greater than 15 cm. Hot spot and slit disappeared when calculation grid size changed to 2.5 mm.

We observed a hot spot and a split in all machines when using calculation grid size of 5 mm for cone size greater than 15 cm and electron beam energy greater than 16 MeV (Fig. 10). The hot spot and split disappeared when grid size was reduced to 2.5 mm with other parameters unchanged. This observation warrants further investigation and a caution using a calculation grid size of 5 mm for final clinical calculation.

## IV. CONCLUSIONS

Agreement between any measurement and Monte Carlo calculation can be affected by both uncertainties associated with measurement and the stochastic nature of the Monte Carlo algorithm. In this paper we reported a systematic study of the differences between the measured data in homogeneous phantom and the Eclipse eMC dose calculation result for four Varian Clinac 21EX linear accelerators in our clinic using the same standard measurement instrument, data acquisition procedure, and beam data fitting procedure. Investigation of the accuracy of eMC algorithm in heterogeneous phantom is beyond the scope of this paper. Various factors, e.g., machine, electron beam emery, electron cone/cutout size, location of the measurement point, and eMC dose calculation grid size, were analyzed statistically. Based on our investigation, we conclude that eMC algorithm in Eclipse provides acceptable agreement between calculation and measurement under most clinical situations using 3%/3 mm criteria when eMC dose calculation grid size is 2.5 mm or smaller and electron cutout size is not smaller than 5×5 cm.

## ACKNOWLEDGEMENT

We are grateful for all the physicists and research associates in the Department of Radiation Oncology at Duke Medical Center who acquired the measurement data.

## APPENDIX ‐ DEFINITIONS OF SOME OF THE PARAMETERS USED IN THIS ARTICLE

Field Width: The width on the profile curve at 50% of the central axis dose.

Penumbra: Distances between 20% and 80% of central axis dose on one side of the profile, averaged over the right and left sides.

$D_{x}$: Dose due to Bremsstrahlung X‐ray. The value is taken to be the dose at $R_{10}+50\;\text{mm}$ on the PDD curve.

$D_{s}$: Surface dose. Defined at 0.5 mm depth on the central axis.

Symmetry: The maximum difference in relative dose between points of equal distance from central axis within the central 80% of the field width. Note that symmetry is sometimes defined as half of the value as defined in here.

Output Factors (OF): The radiation output (dose per MU for a particular cone and/or cutout at $R_{100}$ under the reference open cone) relative to that of the open reference (15×15 cm) electron cone. Note that the definition applies to both the non‐reference open cones and the cutouts.

## Supporting information

Supplementary Material Files
