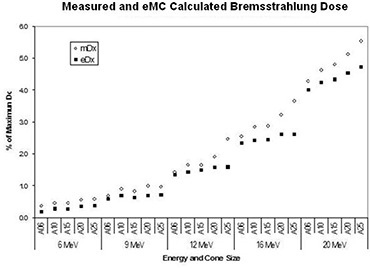# Erratum: “Evaluation of an electron Monte Carlo dose calculation algorithm for electron beams”

**DOI:** 10.1002/j.1526-9914.2008.tb00002.x

**Published:** 2008-06-23

**Authors:** Hu

**Affiliations:** ^1^ Department of Radiation Oncology University of Colorado Health Science Center Aurora CO U.S.A.

JACMP, 9 (3), 1–15 (2008)

In the original article, (Fig. 1a) and Fig. 2 were identical. Below is the corrected Fig. 2.