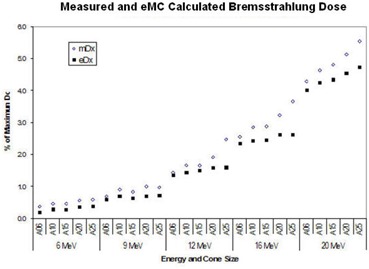# Erratum: “Evaluation of an electron Monte Carlo dose calculation algorithm for electron beams”

**DOI:** 10.1120/jacmp.v10i2.2991

**Published:** 2009-05-13

**Authors:** Hu

**Affiliations:** ^1^ Department of Radiation Oncology University of Colorado Health Science Center Aurora CO U.S.A.

In the original article, Fig. 1(a) and Fig. 2 were identical. Below is the corrected Fig. 2.