# Supplementary figures and images for: Evaluation of an electron Monte Carlo dose calculation algorithm for electron beams
Source: J Appl Clin Med Phys. 2008 Jun 23;9(3):1–15. doi: 10.1120/jacmp.v9i3.2720 (PMC5722292; doi:10.1120/jacmp.v9i3.2720)

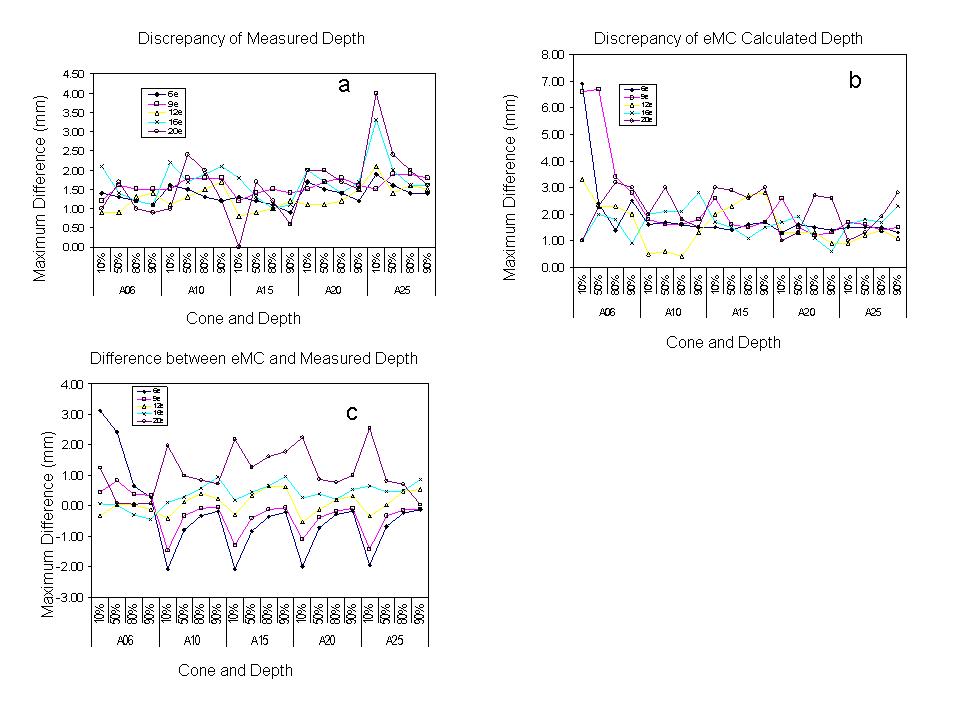

Supplement: Supplementary file 1 — Supplementary Material Files [file ACM2-9-001-s001.jpg]
